# Optimization of physicochemical, textural, and rheological properties of sour cherry jam containing stevioside by using response surface methodology

**DOI:** 10.1002/fsn3.2192

**Published:** 2021-03-06

**Authors:** Ahmad Nourmohammadi, Ebrahim Ahmadi, Ali Heshmati

**Affiliations:** ^1^ Department of Biosystems Engineering Bu‐Ali Sina University Hamedan Iran; ^2^ Department of Nutrition and Food Hygiene Hamadan University of Medical Sciences Hamadan Iran

**Keywords:** jam, rheological properties, stevia, textural properties, TSS

## Abstract

The response surface method (RSM) was used to produce sour cherry jam containing stevia sweetener with favorable physicochemical, textural, and rheological properties. The experiments were designed based on RSM using a central composite design (CCD) with three independent variables: sugar, pectin, and stevia. Sample processing was performed at five levels of sucrose (10%–50%), pectin (0.1%–0.5%), and stevia (0.2%–0.6%) at a constant fruit weight of 300 g. To evaluate the jam, the physicochemical, textural, and rheological properties of the jam were determined and optimized. The concentrations of sucrose, pectin, and stevia had a significant effect on the textural and rheological properties of the jam. The results showed that increasing the concentration of sucrose is the main factor in increasing the soluble solids and the total sugar content of the jam. Pectin had a positive effect on textural characteristics such as hardness and adhesiveness of the jam, while the cohesiveness of the jam decreased with increasing pectin. Herschel–Bulkley model was appropriate for describing the rheological behavior of the stable state of sour cherry jam containing stevia. Decreasing sucrose concentration is accompanied by a decrease in yield stress and consistency index of jam samples due to the TSS reduction. Pectin also had a positive effect on the amount of yield stress and consistency index. Dynamic rheological tests indicated that the sour cherry jam is a weak gel. The predominant viscose behavior and the formation of a dilute solution were evident in the samples with 10% and 20% sucrose concentrations. The most favorable condition for the production of the jam was obtained at a 36.5% sugar, 0.277% pectin, and stevia 0.30%.

## Graphical abstract

Stevia replaced sucrose in jam production. The qualitative properties of the new jam were evaluated and optimized. Using pectin and stevia sweetener can reduce the amount of sucrose in jam.

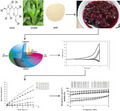

## 1 INTRODUCTION

Jam is a medium‐moisture food that is made by boiling fruit pulp with sugar (sucrose), pectin, acid, and other substances to achieve a sufficient, suitable, and strong consistency to maintain the texture of the fruit (Basu et al., 2013; Belovic et al., 2017). The jam is usually made by mixing 45% by weight of fruit and 55% by weight of sugar. The mixture is then heated until the concentration of it reaches 65% soluble solids. According to EU Council guidelines in 2004, the jam is a mixture of sugars, pulp, or puree of one or more fruits that have reached the gel form with the appropriate consistency (Abid et al., 2018). Typically, traditional jams are widely consumed by several groups of consumers for breakfast, dairy products, bakery products, and confectionery (Igual et al., 2014). With increasing health and wellness concerns and the incidence of obesity, metabolic syndrome, and diabetes, the interest in low‐calorie foods has increased in recent decades (Belovic et al., 2017). Nowadays, given the growth of the food industry, reducing the amount of sucrose in food products with complete or partial replacement of sucrose with alternative sweeteners has become a suitable option for producing low‐calorie or zero‐calorie food products. So, a variety of foods and beverages are produced in which the amount of sugar received and calories produced in the human body is reduced and these products are suitable for obese and diabetic people (Basu et al., 2013). To make jam with lower amounts of sucrose, other carbohydrate and noncarbohydrate sweeteners (such as xylitol, sorbitol, aspartame, acesulfame potassium, cyclamate, stevia, sucralose, or a combination thereof) can be used (Basu et al.,^2011^, ^2013^). Stevia rebaudiana (Bert.) Bertoni is a plant of the family Asteraceae, South America native that has been exploited on large commercial scale since 1970 (Nalesso‐leão et al., 2020). Stevia is a natural sweetener that has become widespread in recent years. Leaf extract of stevia rebaudiana (Bert.) contains large amounts of low‐calorie sweeteners, known as steviol glycosides (Basu et al., 2013; Belovic et al., 2017; Lemus‐Mondaca et al., 2012). Stevia has a high sweetening power. It is resistant to acid and heat and can be used to make jam without altering its taste (Basu et al., 2013). In addition to their sweetening properties, stevia extracts also have antioxidant, antimicrobial, and antifungal activities (Belovic et al., 2017; Lemus‐Mondaca et al., 2012). Sugar is one of the most important ingredients in jam (Javanmard et al., 2012). The removal of sucrose from the jam formulation not only affects the sweetness and taste, but also the crystallization, viscosity structure, moisture, osmotic pressure, fermentation, etc. (Belovic et al., 2017). Fruit jam, which contains an alternative sweetener, should have textural, rheological, and sensory properties similar to traditional and conventional products (Basu et al., 2013; Belovic et al., 2017). Changes in their composition or concentration usually lead to changes in their structure, which are often perceived by the consumer through texture or mouth sensation. Materials and their concentrations affect the quality of the jam, such as appearance (color, texture, and rheology) (Basu & Shivhare, 2010). Recognition of rheological properties is essential for equipment design, processing, product development, quality control, and consumer acceptance (Barbieri et al., 2018). The rheological behavior of jam has been widely studied (Barbieri et al., 2018; Basu & Shivhare, 2010; Basu et al.,^2011^, ^2013^; Igual et al., 2014). Few studies have been conducted on the development of fruit jam using alternative sweeteners (Basu et al.,^2011^, ^2013^; Belovic et al., 2017; Gajar & Badrie, 2002). There are also limited studies on changing the rheological and textural properties of fruit jam with different sweetener concentrations and optimizing the composition of the elements for the jam production process. The response surface method (RSM) is an efficient and modern technique for developing, improving, and optimizing processes that can examine several variables together at the same time and optimize their response. This technique is used in the design, development, and formulation of new products as well as improving the quality of existing products. In this study, the possibility of replacing sucrose with alternative sweetener of stevia at different levels of sucrose, pectin, and stevia was investigated by response surface technique. The effect of replacing sucrose with alternative sweetener on rheological, textural, color, and chemical properties of the sour cherry jam was also investigated.

## 2 MATERIALS AND METHODS

### 2.1 Raw materials

In this research, all the materials needed to produce the jam were provided by Sahar Food Industries, Hamedan. The stevia plant extract was procured from Sahar Food Industries. Low methoxyl pectin (LM) was used to make the jam. Frozen cherries were prepared from the refrigerator of the Sahar factory and placed at room temperature for 6 hr after removing the core. White commercial sugar was used to make jam.

### 2.2 Preparation of jam

First, different levels of sucrose (10, 20, 30, 40, and 50%), pectin (0.1, 0.2, 0.3, 0.4, and 0.5%), and stevia (0.2, 0.3%, 0.4, 0.5, and 0.6%) were weighed by the AND scale (EK30001 model) with measurement accuracy of two decimal places. After measuring the mass, the materials were placed on disposable containers and mixed. The amount of sour cherry fruit mass was kept constant for all samples (300 g), and stevia extract was added to the samples during cooking. The mixture was then transferred to an open stainless steel pot and heated on a low flame. The TSS value was controlled during boiling, and the mixture was continuously stirred during boiling with a glass rod. The flame was extinguished when the TSS reached 65–68˚Brix, and the mixture was poured into 500 g glass jars and cooled at room temperature. To prevent microbial growth, the glass containers were placed in a pot full of boiling water for two hours. The sour cherry jam was produced at different levels of sugar, pectin, and stevia and prepared to measure textural and rheological properties.

### 2.3 Measurement of total soluble solids (TSS)

To measure the value of soluble solids, the Atago PAL‐1 refractometer device made in Japan was used. A drop of jam syrup was placed on the prism of the refractometer after reset to zero by distilled water, and the total soluble solids were read at 20℃. Each sample was measured in three repetitions.

### 2.4 Measurement of total sugar

Fehling titration was used to measure the total sugar of the samples. To the 100‐ml capacity bulb, in which 25 ml of the purified jam solution had previously been poured, 6–10 ml of chloric acid (1 + 3) was added and placed in a water bath at 70℃ for 10 min (acid and heat hydrolyze the disaccharide to monosaccharide). The bulb was then cooled. A few drops of phenolphthalein reagent were added to it and combined with concentrated sodium hydroxide (40%) and then 0.1% normal sodium hydroxide was neutralized until a slightly stable pink color was created. After ensuring color stability, it was made to volume with distilled water. Five milliliters of A Fehling solution and five milliliters of B Fehling solution were poured in 250 ml capacity of Erlenmeyer flask and mixed. A few boiling stones, 3 to 4 drops of Methylene blue, and distilled water (about 20 ml) were added to prevent rapid evaporation. The mixture was heated on a chauffe ballon until it boiled and boiled for about two minutes. Then, the neutralized solution (solution C) was poured into the Burette and placed on top of Erlenmeyer with the help of a base and clamp. During the boiling of the Fehling solutions, the neutralized solution was slowly added to the Erlenmeyer flask until the blue color faded and the ${\mathrm{Cu}}_{2}O$ brick‐red color was created. The volume of the solution used was recorded. The total sugar content was calculated by Equation 1 (William & Latimer, 2005; ISIRI, 2007).

(1) $$N=\frac{F\times 100\times 100\times 100}{V\times 25\times 25}$$

where F is the Fehling factor,^1^ V is the volume of solution used in millimeters, and N is the total sugar (posthydrolysis sugar) in g.

### 2.5 Investigating the textural parameters of jam

**FIGURE 1:**
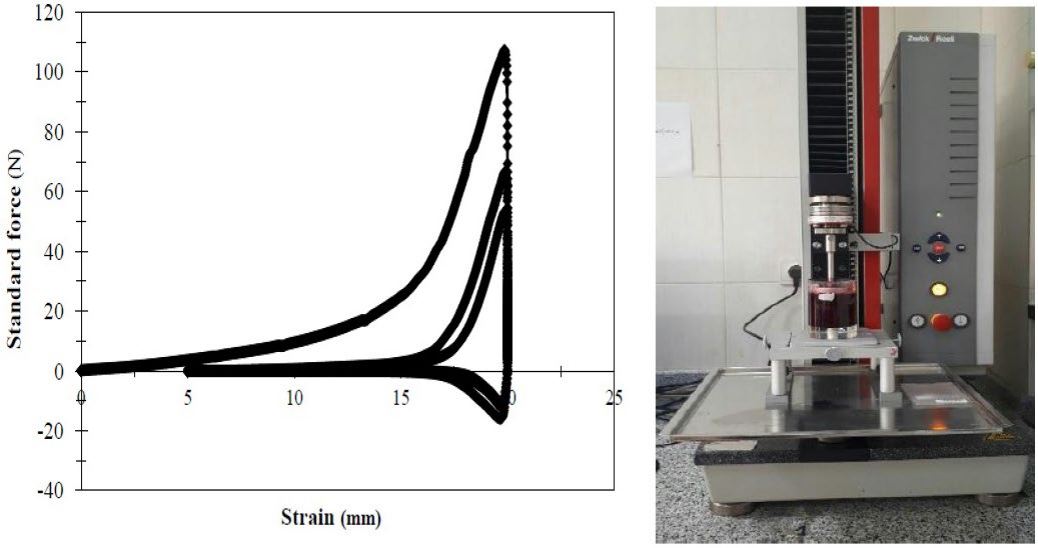
Material Tissue Machine, textural parameters test, An example of the force‐strain diagram of the this test performed on the specimens

Textural characteristics such as hardness, adhesiveness, chewiness, and cohesiveness were assessed using the back extrusion test by ZwickRoell models texture analyzer device (BT1_FR0.5TH.D14 model, Germany) with a loading capacity of 500 Newtons (Figure 1). Jam samples in the same volume (70 cc) were separated for cyclic testing. The diameter of the container was 45.92 mm, and the diameter of the probe was 40 mm. The test was defined in three cycles. Each cycle took 30 s to load and 10 s to unload. The penetration rate of the probe in the jam was 10 mm/s, and the penetration depth was 25 mm. The test was repeated three times for each treatment.

### 2.6 Investigation of rheological properties

The rheological properties of the specimens were measured using Brookfield Rheometer DV‐III with a spindle number CPA‐41Z. Data on apparent viscosity (η_ap_), shear stress (τ), and shear rate (γ ˙) were collected using Rheocalce software (Version T 1.2. 19 Brookfield Engineering Labs Inc.). 2.5 ml of each sample was placed in a cup of the device by a syringe for rheological analysis. All rheological tests were performed at 25℃. The duration of each test was two minutes, and the shear rate varied between 2 and 100 s^‐1^. All experiments were repeated in three steps, and mean analysis was used. The stable state relationship between the shear stress and the shear rate of food was expressed by the Herschel–Bulkley model by Equation (2) (Basu & Shivhare, 2010; Basu et al.,^2011^, ^2013^).

(2) $$\tau =k{\dot{\gamma }}^{n}+\tau_{0}$$

where τ is the shear stress (Pa), τ_0_ is the yield stress (Pa), γ is the shear rate (S^‐1^), K is the consistency index (Pa.s^n^), and n is the flow behavior index (without unit), indicating the rate of deviation from Newtonian behavior.

### 2.7 Dynamic Rheology

The viscoelasticity was measured by the Rheometer (Physica MCR301 model, made by the Austrian company ANTON Paar), equipped with a Peltier Plate temperature adjustment system with a sensitivity of ± 0.01℃. The plate and plate geometry system with a diameter of 20 mm was used, and a gap of 0.5 mm was maintained between the plates during all measurements. The preshearing was performed for 10 s, and the samples were rested for 5 min before the start of the experiment to create thermal equilibrium in the sample and return the additional stresses caused by the loading. The extra sample that came out after loading was carefully cleaned. All rheological measurements (strain sweep, frequency gauges) were performed in triplets at 30℃. The strain sweep was performed at different frequencies for all samples at 30℃ to determine the linear viscoelastic region. The linear viscoelastic range was determined by the strain sweep test in the strain range of 0.001%–1000%. After obtaining the amount of strain in which the material shows the linear structure, frequency sweep experiments were performed at a constant strain of 1% at 30℃. The range of frequencies studied for the test was 0.01–100 Hertz. The obtained oscillating rheological parameters are storage modulus (G'), loss modulus (G"), loss tangent (tanδ), and mixed dynamic viscosity (η*).

### 2.8 Design of experiment

To statistically evaluate the effect of independent variables such as sucrose (x1), pectin (x2), and stevia (x3) on the studied response variables and also to determine the optimal conditions for the process of production of sour cherry dietary jam, the RSM with the central composite design (CCD) was used. The experimental design consists of 20 experiments, consisting of 14‐star points and six central points (with three factors and five levels for each variable) to calculate the repeatability of the method. Each factor in the CCD was studied at five levels (+2, +1, 0, −1, and −2), two axial points (+ α and ‐α), and six repetitions at the central point and the optimal point conditions were determined based on the utility function. The CCD matrix and the results obtained are shown in Table 1.

**TABLE 1:** Experimental design and results for sour cherry jam preparation with stevioside sweeteners at (basis = 300 g sour cherry fruit)

Independent variables						Dependent variable (responses obtained from the experiment)						
Run	Sucrose (% w/w)	Pectin (% w/w)	Stevia (% w/w)	TSS (^0^Bx)	Total sugars (%)	Hardness (*N*)	Cohesiveness (*N*)	Adhesiveness (*N*)	Chewines s (*N*)	Yield Stress (dyn/cm^2^)	K (Pa.s^n^)	*n*
1	10	0.3	0.4	46.2	21.44	112.2	0.28	33.08	20.53	3.08	0.28	0.91
2	20	0.4	0.3	52.36	22.26	98	0.33	36.21	17.97	6.03	4.54	0.80
3	20	0.2	0.3	48.03	22.70	129.2	0.25	24.41	16.71	4.32	1.18	0.88
4	20	0.4	0.5	49.36	28.40	90	0.22	53.96	14.83	5.56	4.39	0.79
5	20	0.2	0.5	47.36	29.31	81.6	0.34	16.11	18.16	4.23	1.96	0.85
6	30	0.3	0.4	55.13	42.70	91.5	0.21	23.04	8.87	10.43	3.99	0.87
7	30	0.3	0.2	57.36	53.38	81.3	0.35	21.3	15.86	12.00	8.82	0.75
8	30	0.3	0.4	56.2	42.89	84.21	0.21	34.36	8.23	8.28	3.87	0.81
9	30	0.1	0.4	55.43	48.74	38.6	0.30	5.47	2.56	10.24	1.50	0.91
10	30	0.5	0.4	53.8	47.56	45.4	0.30	58.74	7.69	13.00	8.82	0.75
11	30	0.3	0.4	55.7	44.30	88.72	0.20	23.48	7.45	10.21	3.43	0.83
12	30	0.3	0.4	53.8	41.29	79.23	0.19	31.93	9.32	10.32	3.56	0.84
13	30	0.3	0.4	53.65	41.14	101.2	0.22	39.11	10.56	10.89	3.71	0.82
14	30	0.3	O.6	53.8	45.64	79.7	0.38	26.77	22.53	10.59	3.52	0.82
15	30	0.3	0.4	54.2	42.48	95.42	0.21	37.26	8.75	9.28	3.78	0.82
16	40	0.2	0.5	60.53	53.49	51	0.30	3.51	24.35	11.76	3.18	0.88
17	40	0.4	0.5	62.61	55.80	107.2	0.28	35.74	21.99	17.87	2.65	0.88
18	40	0.4	0.3	61.3	53.85	62.5	0.29	37.46	11.28	26.21	6.58	0.84
19	40	0.2	0.3	62.23	58.18	75.8	0.26	17.13	15.60	14.66	11.56	0.76
20	50	0.3	0.4	66.1	61.27	95.1	0.30	38.04	27.11	24.79	7.91	0.85

### 2.9 Optimization

In this study, to determine the optimal conditions for the production of sour cherry jam with stevia sweetener, the values of hardness, consistency index, yield stress, and TSS were examined in maximum values, while total sugar variables, adhesiveness, chewiness, and flow index were considered at the minimum level and all three independent variables (sucrose, pectin, and stevia) and the variables of response, cohesiveness were considered at in range.

### 2.10 Statistical analysis

In this study, Design‐Expert software (version 10.0.0, Statease Inc., Minneapolis, USA) was used to analyze the data and optimize the jam production process. The correlation coefficient and the lack‐of‐fit criterion were used to determine the significance of regression equation models (Table 2). Analysis of variance (ANOVA) was performed on linear or single second‐order regression coefficients, main interaction, and double effects. The significance of all terms in a polynomial was determined statistically by calculating the value of P at the probability level of 0.01 and 0.05. The complete second‐order equation for each jam variable using the RSM was obtained as follows Equation (3):

**TABLE 2:** Regression coefficients of coded factors and ANOVA data for the responses in the optimization of sour cherry jam preparation with stevioside sweeteners

Coefficients	TSS (^0^Bx)	Total sugars (%)	Hardness (*N*)	Cohesiveness (*N*)	Adhesiveness (*N*)	Chewiness (*N*)	Yield Stress (dyn/cm^2^)	K (Pa.s^n^)	*n*
Intercept	54.84	41.61	91.48	0.21	31.03	9.48	10.43	3.68	0.84
X1	5.65**	12.39**	−8.53**	1.98E−003	−1.68^ns^	1.17^ns^	5.86**	1.70**	−4.6E−003^ns^
X2	0.85*	−0.36^ns^	2.11^ns^	−2.8E−003^ns^	13.05**	0.095^ns^	1.88**	0.93*	−0.023*
X3	−0.52^ns^	0.066^ns^	−2.43^ns^	3.79E−003^ns^	0.32^ns^	1.94*	−1.08**	−1.39**	0.016*
X1X2	−0.77^ns^	−0.082^ns^	8.21*	5.3E−003	0.36^ns^	−0.58^ns^	1.83**	−1.41*	0.029*
X1X3	0.29^ns^	−1.94^ns^	9.44*	6.1E−003^ns^	−3.10^ns^	2.64*	−1.34*	−1.62**	0.026*
X2X3	0.21^ns^	0.77^ns^	13.64**	−0.032*	4.74*	−0.33^ns^	−0.73^ns^	0.44^ns^	−7.2E−003^ns^
X1^2^	0.37^ns^	−0.58^ns^	4.12*	0.020**	0.76^ns^	4.05**	0.87**	0.067^ns^	0.012^ns^
X1^2^	0.23^ns^	1.11^ns^	−11.30**	0.023**	−0.11^ns^	−0.63^ns^	0.041^ns^	0.33^ns^	1.2E−005^ns^
X3^2^	−0.012^ns^	0.45^ns^	−1.67^ns^	0.038**	−2.12^ns^	2.89**	0.042^ns^	0.59*	−0.012^ns^
Model (p‐value)	<0.0001	0.0004	0.001	<0.0001	0.001	0.0008	<0.0001	0.001	0.016
Lack of fit (*p*‐value)	0.1879	0.0006	0.1792	0.0381	0.5414	0.0050	0.0003	<0.0001	0.1430
R^2^	0.96	0.93	0.88	0.93	0.87	0.89	0.97	0.88	0.79

Note

X_1_: sucrose.

X_2_: pectin.

X_3_: stevioside.

* Significant at *p* ≤ .05.

** Significant at *p* ≤ .01. ns not significant.

Where Y is the response of the dependent variables (TSS, total sugar, hardness, cohesiveness, chewiness, adhesiveness, yield stress, consistency index, and flow index) βo is the width of origin, βi, βj, and βij are the linear coefficients, second‐order, and interaction of the relationship, respectively, Xi and Xj are independent variables.

(3) $$y=\beta_{0}+\sum_{j=1}^{k}\beta_{i}x_{i}+\sum_{i<j}\sum \beta_{\mathrm{ij}}x_{i}x_{j}+\sum_{j=1}^{k}\beta_{\mathrm{ij}}x_{j}^{2}$$

## 3 RESULTS AND DISCUSSION

### 3.1 TSS

TSS is an important factor used to evaluate the quality of the jam. The TSS values of the jam samples in the specified levels of sugar, pectin, and stevia are shown in Table 1. Based on the results of Table 2, it was observed that the linear effect of sucrose (*p* < .01) and pectin (*p* < .05) on the amount of TSS in the sour cherry jam was significant. As the percentage of sucrose and pectin increased, the TSS value of sour cherry jam increased, but changes in stevia concentration did not affect the TSS amount of jam. Pectin forms a biopolymer gel network when sucrose is present as a common solution, and changes in the concentration of materials significantly affect the network structure and integration of the pectin biopolymer chains in the system (Basu et al., 2013). Sucrose plays an important role in improving TSS of jam, and its deficiency reduces the soluble solids in the jam, resulting in a diluted syrup with low product consistency. The results showed that different amounts of stevia had no significant effect on the amount of TSS in jam samples. One of the main reasons why these parameter do not affect TSS is the very low values of these compounds in jam formulations (Yousefi et al., 2012). The amount of total soluble solids (TSS) in most samples was in the lower range compared with the values obtained for commercial products reported by CODEX (2009).

### 3.2 Total sugar

The total sugar content of sour cherry jam was in the range of 21.447% to 61.272%. According to the results of the fitted model to the experimental data, it was observed that the only linear effect of sucrose (X1) on the total sugar content of sour cherry jam was significant (*p* < .01) (Table 2). The results showed that the changes in stevia at the specified levels did not affect the total sugar content of sour cherry jam and the major changes in sugar were related to increasing or decreasing the amount of sucrose (Table 1). Belovic et al. (2017) reduced total sugar and carbohydrates of the jam by replacing fructose and stevia with sucrose and making it suitable for diabetics.

### 3.3 Texture

#### 3.3.1 Hardness

**FIGURE 2:**
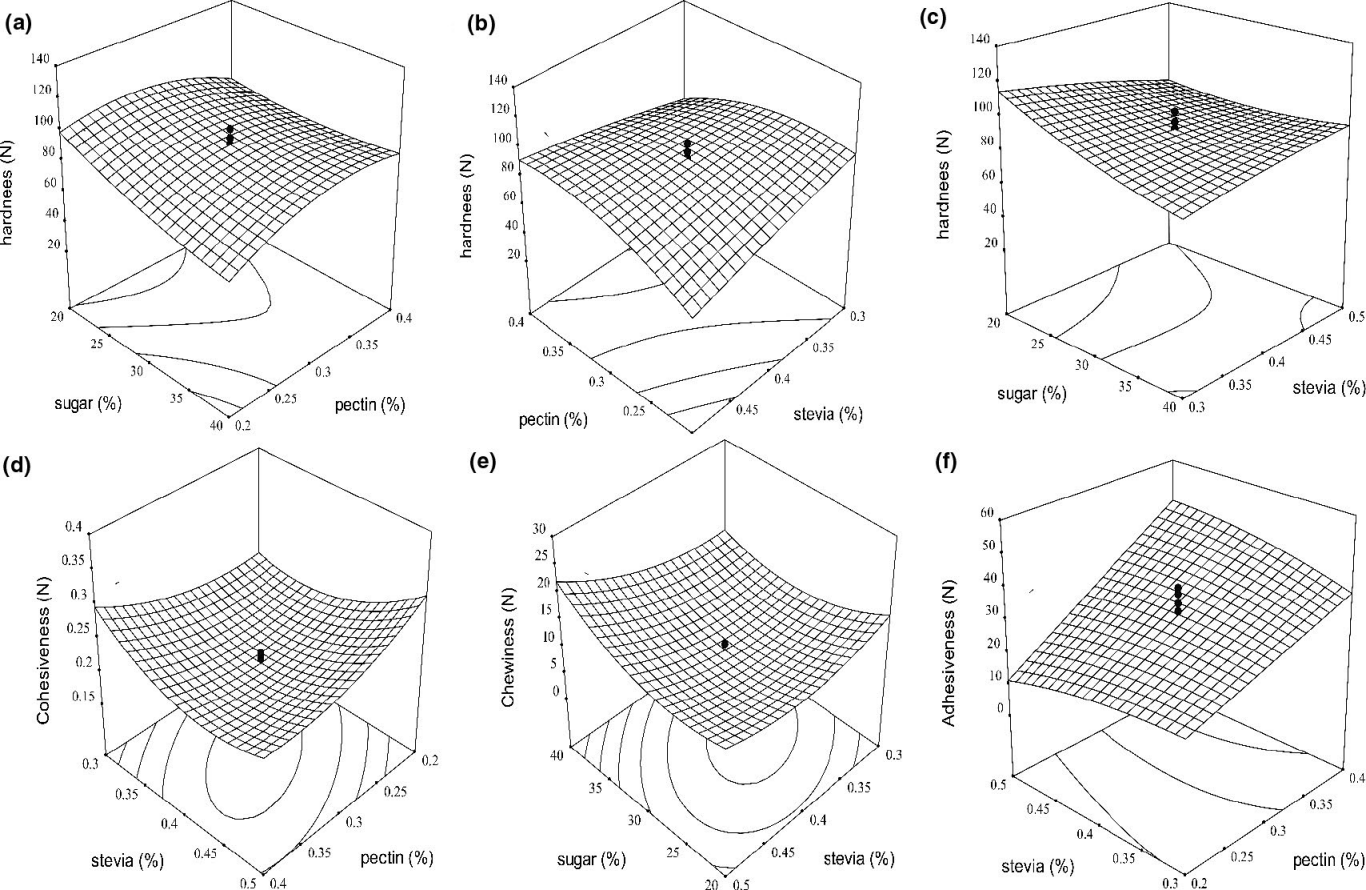
Response surface plot of the effects of sucrose, pectin and Stevioside on hardness (A, B, C), Cohesiveness (D), Chewiness (E), and adhesiveness (F) of sour cherry jam preparation with stevioside sweeteners

Hardness is defined as the amount of force required to achieve a certain amount of deformation and is a common parameter in determining jam texture (Mousavi et al., 2019). In the sensory analysis, hardness is the force required to compress food between teeth in the first bite (Garrido et al., 2015). The highest hardness (129.2 N) was obtained in 20% sucrose, 0.2% pectin, and 0.3% stevia. The lowest hardness (38.6 N) was obtained in 30% sucrose, 0.1% pectin, and 0.4% stevia (Figure 2A,B,C). The results of the analysis of variance showed that the interaction effect of sucrose and pectin (X_1_X_2_), sucrose and stevia (X_1_X_3_), and pectin and stevia (X_2_X_3_) on the hardness of sour cherry jam was significant at the levels of (*p* < .05), (*p* < .05), and (*p* > .01), respectively. As the concentration of pectin increased, the hardness of sour cherry jam increased. Pectin is a gelling agent that is responsible for the formation of gels during the formation of jam in the presence of sucrose. As a result, the number of active polymer chains in the gel network increases by increasing the concentration of pectin, so the structural network inside the gel becomes harder (Basu & Shivhare, 2010). It can also be seen that increasing the concentration of fruit in the jam increases the hardness, which can be attributed to the increase in the inherent pectin content of the fruit. According to the results, as the sucrose and stevia increased simultaneously, the hardness of the jam decreased (Figure 2C). The reason for the decrease in hardness can be ascribed to the weakening of pectin gel and the release of a large amount of water in the jam, which makes it softer and reduces the hardness of the jam. Basu et al. (2013) reported that high sugar concentrations destabilize the hydrogen bond between polyhydric sucrose and water molecules leftover from the hard gel network of pectin. Generally, the results showed that adding pectin had a positive effect on hardness, while high concentrations of sucrose and stevia reduced the hardness of the jam.

#### 3.3.2 Cohesiveness

Cohesiveness indicates the internal resistance of food structures and means the ability to combine components of a product (Azari‐Anpar et al., 2017). Based on the results of the fitted model to the experimental data, it was observed that the interaction of pectin and stevia (X_2_X_3_) on the cohesiveness of sour cherry diet jam was significant (*p* < .01). The cohesiveness of the sour cherry jam sample varied from 0.19 to 0.38 (Figure 2D). Increasing pectin concentrations reduced cohesiveness of sour cherry jam. This means that as the pectin concentration increased, the jam texture became brittle (Abid et al., 2018).

The reason for this is that when too much elastic stress is applied to a strong gel, the permanent and temporary bonds are irreversibly destroyed and the gel is broken, so shows poor adhesiveness. But the samples with weaker gel structures had more cohesiveness. The weak gel structure is mainly created by temporary bonds that may be partially corrected after the applied stress has been removed, thus show high cohesiveness (Raphaelides et al., 1996). According to the results, increasing the pectin factor reduces the cohesiveness of the jam.

#### 3.3.3 Chewiness

Chewiness or solubility is a property of foods that determine the ability to chew the product. The chewiness is defined as the time (or energy) required to chew the sample to convert it to the ready‐to‐swallow state (Azari‐Anpar et al., 2017). Higher value specimens are more resistant to chewing force; as a result, their chewiness is higher (Culetu et al., 2014). ANOVA results showed that the interaction of sucrose and stevia on sour cherry jam was significant (*p* < .05). The highest chewiness (27.11 N) was obtained in 50% sugar, 0.3% pectin, and 0.4% stevia. Also, the lowest chewiness (2.56 N) was obtained in 30% sugar, 0.1% pectin, and 0.4% stevia (Figure 2E). With the increase in sucrose, the chewiness of sour cherry jam increased, but the increase in stevia reduced the chewiness and softening of the jam texture. The gradual increase in sugar concentration seems to reduce the amount of water in the pectin–sugar and acid mixture to some extent, thus reduces the possibility of hydrogen bonds and other possible causes, thereby reduce the softness of the jam texture and increase the chewiness rate. Increases. Sugars generally reduce water activity, which causes pectin interactions instead of pectin–water interactions (Belovic et al., 2017). The above results showed that sucrose is the main cause of increased chewiness and improves the texture quality of the samples.

#### 3.3.4 Adhesiveness

Adhesiveness indicates the work needed to extract the pressure probe from the sample. In the sensory analysis, adhesiveness is the work required to overcome the gravitational forces between the surface of the food and the surface with which the food is in contact (tongue, teeth, palate) (Garrido et al., 2015). The highest adhesiveness was (58.74 N) in 30% sucrose, 0.5% pectin, and 0.4% stevia. Also, the lowest adhesiveness in the sour cherry jam was (3.31 N) in 40% sucrose, 0.2% pectin, and 0.5% stevia. The results of the analysis of variance indicate that the linear effect of pectin (x_2_) on the adhesiveness of the jam was significant (*p* < .01), and also the interaction of pectin and stevia (x_2_x_3_) on the degree of adhesiveness of the jam was significant (*p* < .05) (Table 2). According to the results, the pectin factor had a positive effect on the degree of adhesiveness, so that increasing the percentage of pectin increased the adhesiveness of the jam. The results of the present study were consistent with the findings of Abid et al. (2017) (Figure 2F). The results also showed that the adhesiveness decreased with increasing concentration of stevia. The results also showed that the change in stevia concentration had no significant effect on the adhesiveness of the jam; however, at high levels of stevia concentration, the adhesiveness of the jam decreased. Basu et al. (2013) reported that by increasing the concentration of stevia and replacing it with sugar, the adhesiveness of jam decreases, which is consistent with the results of this study.

### 3.4 Steady‐state rheology

**FIGURE 3:**
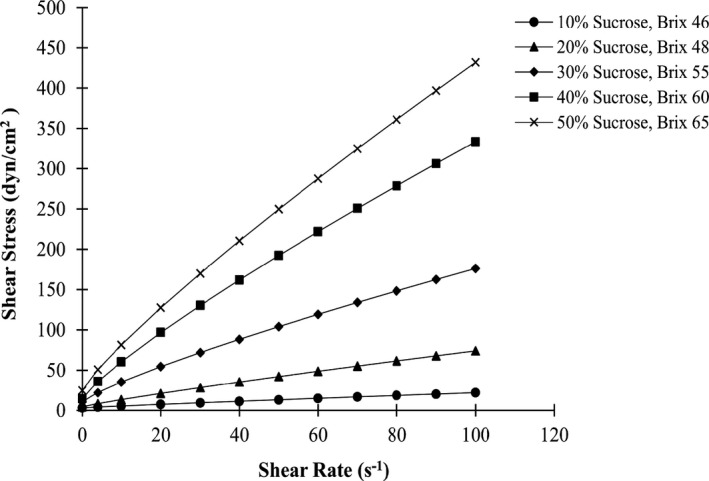
Steady‐state rheogram of sour cherry jam at selected sugar concentration *Herschel‐Bulkley model (R^2^ = 0.996)*

**FIGURE 4:**
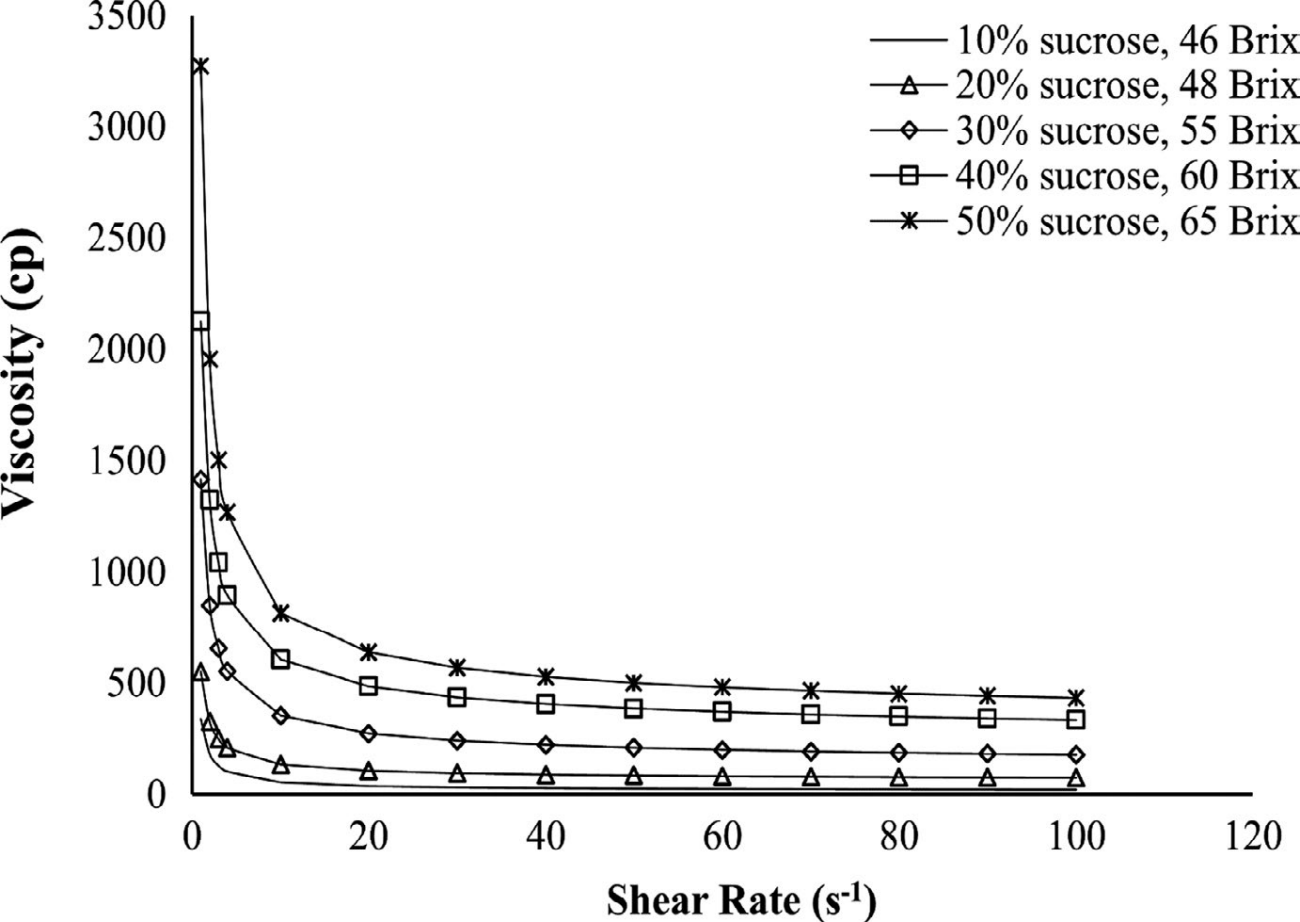
Viscosity – shear rate rheogram at different sugar levels and different TSS values

The rheological behavior of the steady state of the sour cherry jam was shown at different levels of sucrose and different concentrations of TSS (Figure 3). Based on the figure, it was found that the Herschel–Bulkley model predicted the rheological behavior of sour cherry jam at different levels of sucrose, pectin, and stevia. The jam samples showed the non‐Newtonian pseudoplastic flow behavior with yield stress. The flow curves showed the difference between the samples prepared with different concentrations of sucrose. According to the figure in the same shear rate range, samples with high‐sucrose concentrations had higher shear stress values than samples with low concentrations. This means that jams with high‐sucrose are more resistant to applied shear stress than other samples. Figure 4 shows the viscosity changes with increasing shear rate. Increasing the shear rate caused the nonlinear reduction in the viscosity curve, meaning that the specimens had shear‐thinning behavior. Also, according to the flow curves, it was found that the samples with higher sucrose concentration and TSS value have higher viscosity. Shear‐thinning behavior was also observed in the jam of other fruits such as mango (Basu & Shivhare, 2010), apple (Guo et al., 2017), and gabiroba (Barbieri et al., 2018), which is determined by the flow curve. Herschel–Bulkley model was appropriate for the rheological behavior described by the flow curves, and the value of R^2^ for it was 0.996, which is similar to the results of mango jam (Basu & Shivhare, 2010), with R^2^ values between 0.818 and 0.996 and apple jam (Guo et al., 2017), with R^2^ values from 0.891 to 0.998. Parameters such as yield stress (t_0_), consistency index (k), and flow behavior index (n) are determined using the Herschel–Bulkley model, which indicates the rate of deviation from Newtonian behavior (Barbieri et al., 2018).

#### 3.4.1 Yield stress

**FIGURE 5:**
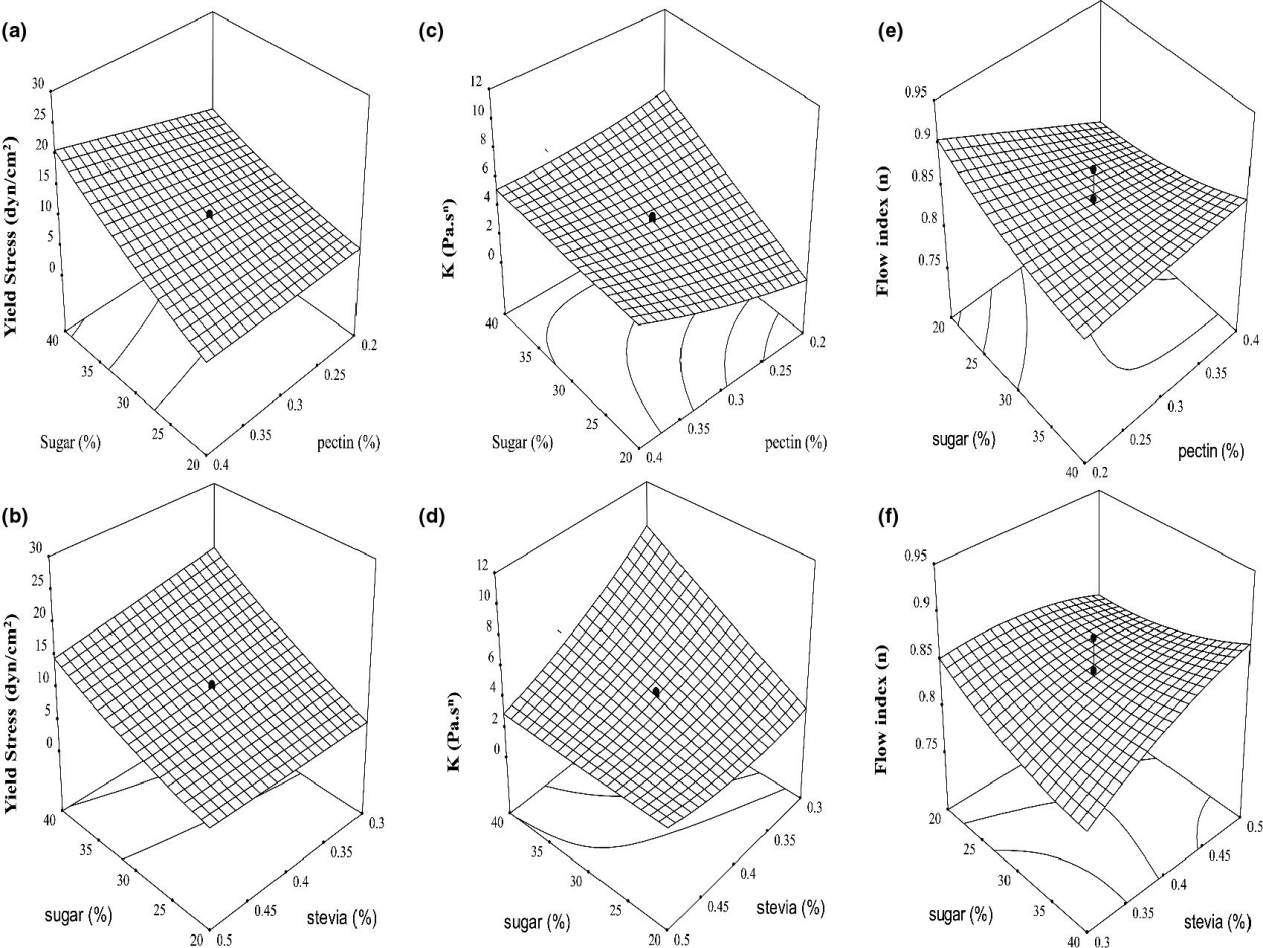
Response surface plot of the effects of sucrose, pectin, and Stevioside on yield stress (A, B), consistency index (C, D), and flow index (E, F) of sour cherry jam preparation with stevioside sweeteners

Yield stress is the minimum shear stress required for fluid flow. Above the yield stress point, the fluid begins to flow (Rao, 2010). The presence of yield stress during the flow of a material indicates that there is an intersecting structure or other reciprocal structure that must be broken before the fluid can flow at the appropriate rate (Barbieri et al., 2018). The highest yield stress (26.21 dyn/cm^2^) was obtained in 40% sucrose, 0.2% pectin, and 0.3% stevia (Table 1). Also, the lowest yield stress (3.08 dyn/cm^2^) was obtained in 10% sucrose, 0.3% pectin, and 0.4% stevia. The results of the analysis of variance showed that the linear effect of sucrose, pectin, and stevia on the yield stress of sour cherry jam was significant (*p* < .01). Also, the interaction of sucrose and pectin (x_1_x_2_) and sugar and stevia (x_1_x_3_) on the yield stress of sour cherry jam was significant at the level of *p* < .01 and *p* < .05, respectively (Table 2). The negative coefficient of stevia in the fitted model to the experimental data using the RSM indicates the negative effect of this variable on the yield stress. Basu et al. (2013) reported that at the specified level of stevia, the values of yield stress decreased with increasing stevia. Figure 5A,B shows the interaction effect of sucrose, pectin, sugar, and stevia on the yield stress of sour cherry jam, respectively. According to the figure, as the concentration of sucrose and pectin increased simultaneously, the amount of jam yield stress increased. Also, increasing the concentration of stevia slightly reduced the yield stress. It is predicted that as the concentration of sugar increases, the amount of soluble solids in the jam increases (Guo et al., 2017), as a result of which the suspended solids in the fluid increase and the fluid moves away from Newtonian behavior and the yield stress increases. Few previously published reports also (Basu & Shivhare, 2010; Basu et al., 2011; Belovic et al., 2017) attributed the increase in yield stress to the increase in sugar and TSS, which were consistent with the results of the study.

#### 3.4.2 Consistency index

The consistency index (K) is a parameter of matter that reflects the internal structure of food fluids. K is a parameter similar to viscosity that indicates shear strength (Guo et al., 2017). The value of the consistency index of sour cherry jam varied from 0.28 (pa.s^n^) to 11.56 (pa.s^n^) (Table 1). According to the results of the model fitted to the experimental data in Table 2, it was observed that the linear effect of sugar (x_1_), pectin (x_2_), and stevia (x_3_) on the consistency index of the sour cherry jam was significant. Also, the interaction of sucrose and pectin (x_1_x_2_) and sucrose and stevia (x_1_x_3_) on the consistency index of the sour cherry jam was significant at the level of *p* < .05 and *p* < .01, respectively. Figure 5C,D shows the interaction of sucrose and pectin with sucrose and stevia on the consistency of the sour cherry jam index. According to the figure, with increasing sucrose and pectin, the consistency of the jam increased. Also, with increasing stevia concentration in the samples, the jam consistency index decreased (Basu et al., 2013). The amount of consistency varies according to their formulation compounds (Falguera et al., 2010). Basu and Shivhare (2010) reported that the difference between k and *n* values with changes in sugar and pectin levels was not significant. Some R^2^ values from their research were at least 0.818, while Belovic et al. (2017) reported that the main reason for the increase in jam consistency is the increase in sugar. Figure 5C,D shows the interaction of sucrose and pectin with sucrose and stevia on the consistency index of sour cherry jam. Guo et al. (2017) and Basu et al. (2011) also reported that the increase in soluble solids is the main factor in increasing the consistency index of low‐calorie jam. They also reported that pectin does not affect the k value. According to the results, it can be said that sugar and pectin have a positive effect on the consistency index.

#### 3.4.3 Flow index

The flow index is a parameter that expresses the internal structure of food fluids (Guo et al., 2017). n indicates fluid deviation from Newtonian behavior. Based on the results, it was found that the linear effect of pectin (x_2_) and stevia (x_3_) on the flow index of the sour cherry jam was significant (*p* < .05) (Table 2). The values of the coefficient of determination (R^2^), the lack‐of‐fit factor, and the coefficient of variation for hardness were obtained equal to 0.79, 0.1430, and 3.54, respectively, which indicates that the model obtained by the RSM is not suitable for the flow index stress. Figure 5E,F shows the interaction of sucrose, pectin, sucrose, and stevia. According to the figure, it can be concluded that with increasing the concentration of sucrose and pectin, the flow index decreased. Also, with the increase in stevia, the flow index increased. It seems that with increasing sugar concentration, the rate of deviation from Newtonian flow in jam is higher and samples with high sugar content had a lower flow index (Belovic et al., 2017). Basu and Shivhare (2010) reported that the difference in *n*‐value with changes in sugar and pectin levels was not significant. Some of the R^2^ values from their research were at least 0.818, which, given the low value of R^2^ obtained (0.79) for the flow index of the sour cherry jam, the results were consistent with the obtained values of the research. In another study, Basso et al. Basu et al. (2013) observed that n increased with decreasing TSS values, indicating Newtonian behavior of the jam. According to the low R^2^ values, it can be found that sugar, pectin, and stevia factors did not have much effect on the flow index of jam.

### 3.5 Dynamic Rheology

**FIGURE 6:**
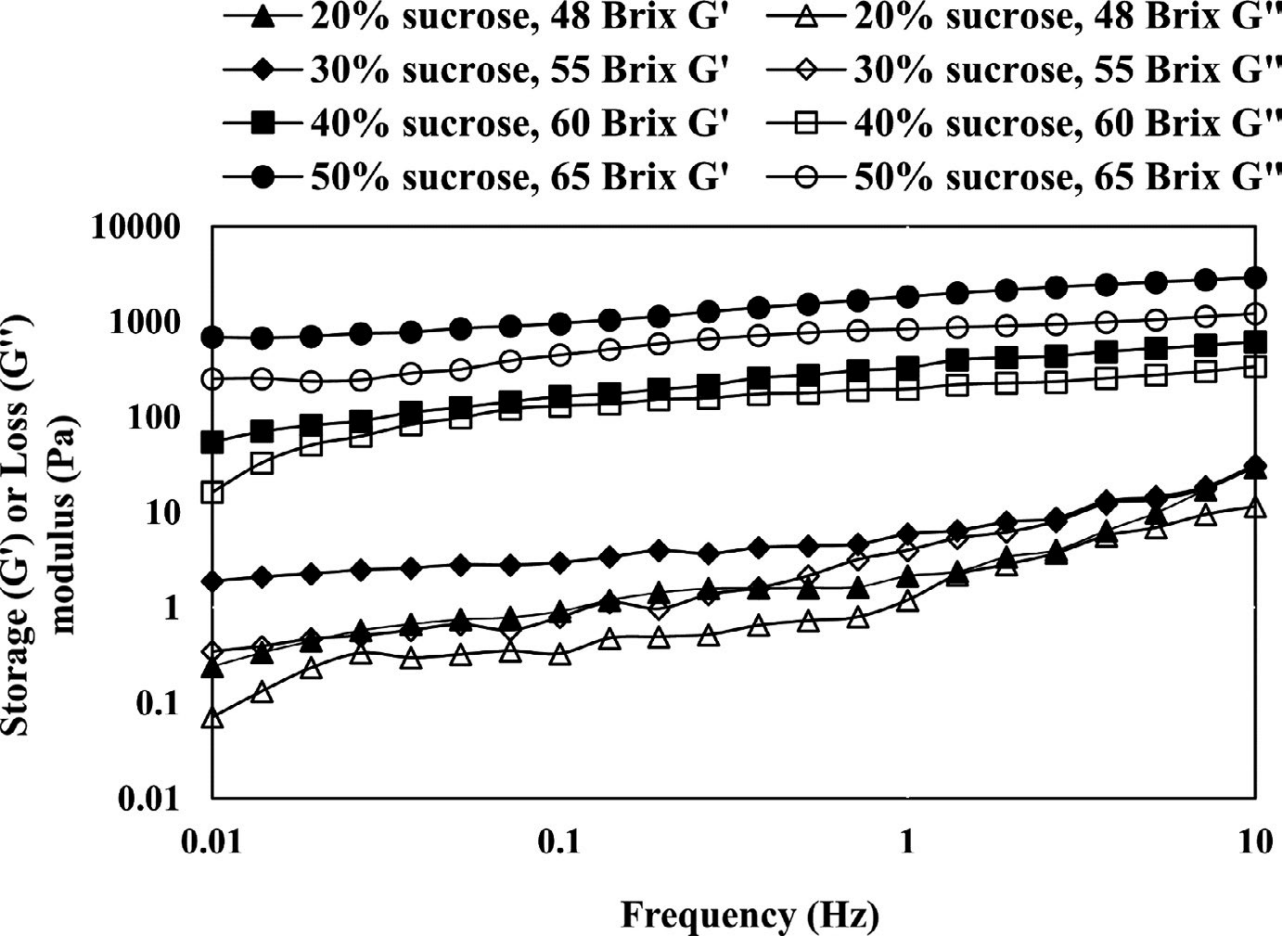
Storage and loss moduli of sour cherry jam at different sugar levels (50%, 40%, 30%, 20%) and different TSS values (65brix, 60brix, 55brix, 48brix)

**FIGURE 7:**
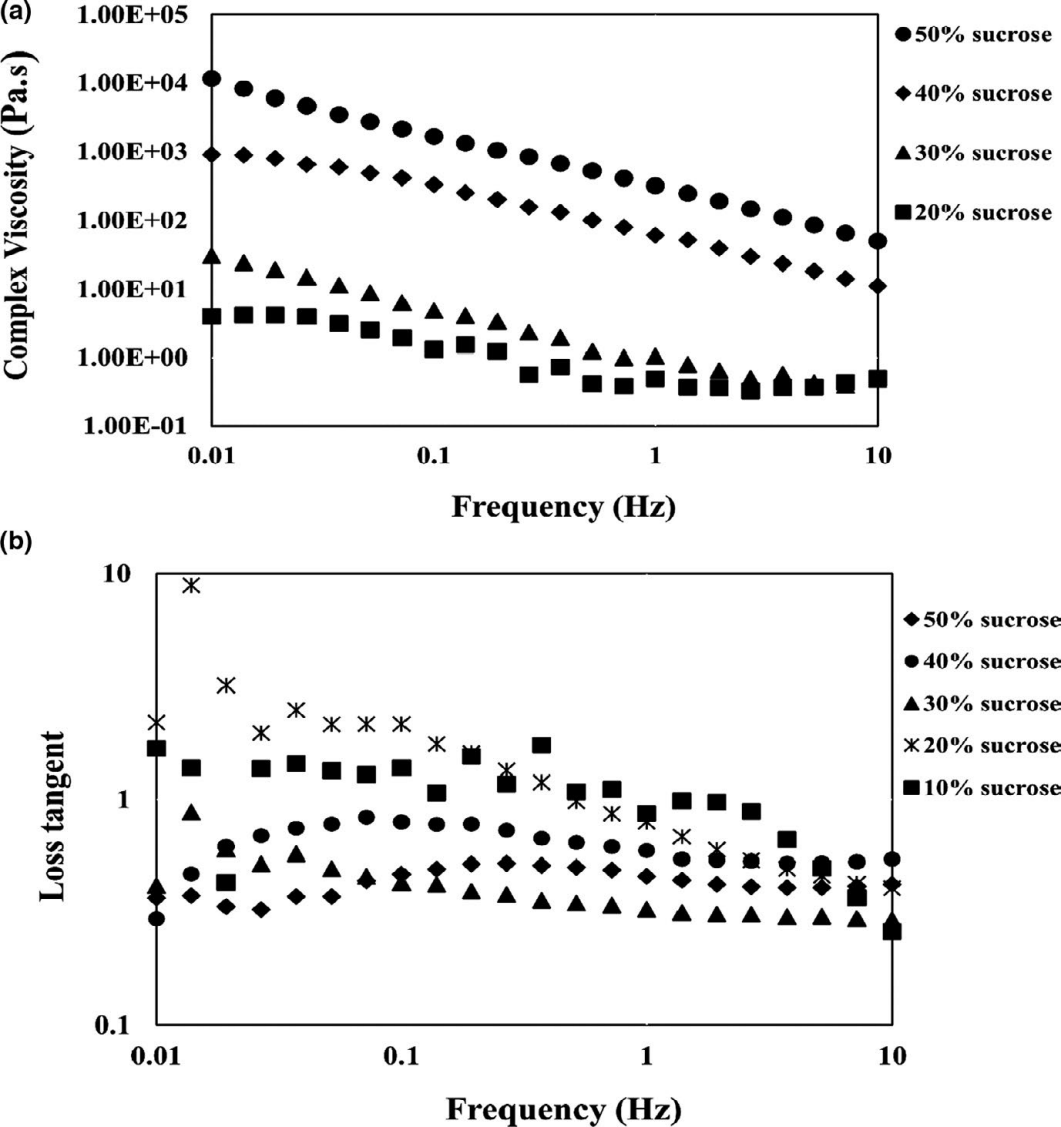
Variation of complex viscosity at different levels of sugar (A), Loss angle as a function of frequency in at selected sucrose levels (B)

A frequency sweep test was used to determine the viscoelastic properties of jam samples. Frequency sweep is the most common oscillation test. In this test, the oscillation amplitude of stress or input strain is kept constant, while the frequency is increased. In this test, the storage modulus (G') and the loss modulus (G") were examined. The values of the storage modulus and the loss modulus of the sour cherry jam samples at different levels of sucrose and the TSS are shown as a function of frequency (Figure 6. It can be seen that the slope of the sample at 48 °BX is faster than the other two samples (60 and 65°BX). The different slope values indicate the different nature of the bond in the samples. Samples with low concentrations of sucrose and low TSS (48 and 55°BX) had faster ranges and behavior such as liquids, and jams with higher TSS and sucrose values had lower ranges and behaviors such as soft solids. The amount of storage modulus (G') of jams in the applied frequency range (0.1–100 Hz) in some points was lower than in the loss modulus (G"), and several intersecting points were observed. As a result, some samples are dilute. The samples with high‐sucrose concentrations (40 and 50%) can form weak gels, and this ability increases with increasing pectin content. Figure 6 shows the dependence of the storage modulus and the loss modulus at different sucrose levels and the specified frequency range. The values of the loss and storage modulus increased with increasing sucrose concentration. Also at low sucrose concentrations, the storage and loss modulus intersected at points, and the amount of the loss modulus exceeded the storage modulus, indicating a diluted solution for jam. For high‐sucrose concentrations (40% and 50%), the G' value at any given point of the variable frequency tests was greater than that of G" (Figure 6), indicating the viscoelastic behavior of sour cherry jam. In general, sucrose acts as such a sluice material on the fruit jam that disturbs the balance between water and pectin (Basu et al., 2011). Gradually increasing the sucrose concentration slightly reduces the water in the pectin–sucrose–acid mixture, and as a result, it reduces the likelihood of hydrogen bonding and the possible association of water with the pectin polymer chain (Bayarri et al., 2004). Similar results reported for different food systems, including Basu et al. (2011) for mango jam with sorbitol substitute, Basu et al. (2013) for mango jam with stevia substitute, Rangriz et al., (2015) in mayonnaise sauce, and Díaz‐Ocampo et al. (2014) for Borojo jam. Pectins are also widely used in jam formulations to enhance the strength and power of gels, with a strengthening effect on the structure of the jam (Barbieri et al., 2018; Basu & Shivhare, 2010; Garrido et al., 2015). The connecting areas of the pectin network are stabilized by various interactions, which can affect network structure and viscoelastic behavior (Barbieri et al., 2018). Another factor obtained from this test is the complex viscosity (η*). In this regard, the trend of its changes in the sample with respect to the angular frequency is shown in Figure 7A. The complex viscosity is a measure of the hardness of the material (Niknam et al., 2018). As can be seen, with increasing frequency, the complex viscosity decreases such that the dilution behavior of the samples is quite obvious. It should be noted that at all frequencies, the sample with 50% sucrose had the highest complex viscosity. The results showed that with increasing sugar concentration, the value of complex viscosity in the samples increased. The slope of the complex viscosity diagram (η*) versus the angular frequency is one of the important methods for determining the strength of the gel. High levels of this slope indicate the elastic behavior (gel) of the jam, which in turn depends on various parameters, including the concentration of sucrose and pectin in the jam. It should be noted that the slope of the complex viscosity diagram in samples with higher sucrose percentage (40 and 50%) was higher than other jam samples, indicating elastic behavior, and the ability to form weak gels by these samples. In this regard, Basu et al. (2013), Basu et al. (2011), Bayarri et al. (2004), as well as, achieved similar results on mango jam. Another factor investigated in this study was loss tangent (tanδ). This factor indicates which rheological property (viscose or elastic) is predominant in the viscoelastic material. In other words, it indicates the ratio of energy dissipated as viscosity (G") to stored energy as elastic (G'). As shown in Figure 7B, the value of loss tangent in samples with a low percentage of sugar (10 and 20%) was higher than one, indicating the predominant viscous behavior in these samples. Also, in jams with a high percentage of sugar (40 and 50%), the amount of tanδ was less than one, which indicates that the elasticity is higher than viscosity. Among all the samples, the lowest value of loss tangent was observed in the sample with 50% sugar, which indicates that this sample has more elasticity than other samples. In this regard, Basu et al. (2013) and Basu et al. (2011) achieved similar results, so that in all samples of mango jam produced by them, the amount of tanδ was less than one. Basu et al. (2011) reported that with increasing TSS, the value of loss tangent decreased, indicating the increase in elastic behavior in the material, but in the selected concentrations of sucrose, the value of loss tangent did not differ much.

### 3.6 Optimization

Optimizing the production process of sour cherry jam containing stevia is an important step in achieving the desired quality products in terms of nutritional value, marketability, durability, and economy. The RSM and the utility function were used to optimize all response variables at the same time. Textural, physicochemical, and rheological properties for sour cherry jam produced with stevia sweetener were optimized at the same time and based on the objectives in Table 3. Ten solutions were obtained to determine the optimal conditions with the value of the desirability index of 55% using the RSM and the utility function (Table 3). The results obtained from Table 3 show that the optimal values obtained were very close to each other, the most favorable condition for the production of the jam was obtained at a 36.5% sugar, 0.277% pectin, and stevia 0.30%. The optimal value for the dependent variables including TSS, total sugar, hardness, cohesiveness, adhesiveness, chewiness, gumminess, springiness, yield stress, consistency index, and flow index with 55% desirability is shown in Table 3.

**TABLE 3:** Optimum value of different variables for the production of sour cherry jam preparation with stevioside sweeteners

Number	Sucros e (%)	Pectin (%)	Stevia (%)	TSS (^0^Bx)	Total sugars (%)	Hardnes s (*N*)	Cohesivenes s (*N*)	Adhesivenes s (*N*)	Chewiness (*N*)	Yield Stress (dyn/cm^2^)	K (Pa.sn)	*n*	Desirability
1	36.50	0.277	0.30	58.97	52.58	83.10	0.246	27.90	11.13	15.74	7.95	0.795	0.55
2	36.48	0.277	0.30	58.95	52.56	83.15	0.246	27.82	11.12	15.69	7.94	0.795	0.55
3	36.48	0.278	0.30	58.96	52.54	83.12	0.246	27.96	11.12	15.75	7.94	0.795	0.55
4	36.46	0.276	0.30	58.94	52.53	83.18	0.246	27.79	11.11	15.66	7.94	0.795	0.55
5	36.38	0.277	0.30	58.90	52.42	83.28	0.246	27.84	11.08	15.63	7.91	0.795	0.55
6	36.62	0.279	0.30	59.04	52.70	82.92	0.247	28.06	11.18	15.89	7.98	0.795	0.55
7	36.45	0.275	0.30	58.93	52.55	83.21	0.246	27.64	11.10	15.59	7.94	0.795	0.55
8	36.48	0.280	0.30	58.95	52.50	83.09	0.247	28.14	11.13	15.83	7.92	0.795	0.55
9	36.59	0.275	0.30	59.02	52.73	83.00	0.246	27.69	11.15	15.71	8.00	0.795	0.55
10	36.34	0.279	0.30	58.87	52.34	83.00	0.246	27.95	11.07	15.65	7.88	0.795	0.55

## 4 CONCLUSION

The present study aimed to produce low‐calorie sour cherry jam by adding stevia sweetener with the lowest caloric content and achieve the desired physicochemical, textural, and rheological properties. To achieve these goals, the RSM was used to optimize the sucrose, pectin, and stevia levels. According to the TSS of the product and also emphasizing the low‐calorie content of the produced jam, the optimal production point for mass production and the study of textural and rheological properties was obtained at 36.5% sucrose, 0.277% pectin, and 0.3% stevia. The jam produced with a concentration of 10%, 20%, and 30% sucrose does not meet the desired textural and rheological properties for sour cherry jam because of the low TSS in the final product. The production of sour cherry jam with the desired soft solid properties was possible only with 40% and 50% sucrose. Herschel–Bulkley model was suitable for describing the steady‐state rheological behavior of sour cherry jam produced using stevia sweetener. Total sugar levels decreased with decreasing sucrose concentration. The dynamic biological rheological tests described sour cherry jam as a weak gel. The predominant viscous behavior and the formation of a diluted solution were evident in the samples with 10% and 20% sucrose. The results showed that pectin and stevia sweeteners could reduce sugar levels in jams. The findings of the present study can be used to prepare low‐calorie cherry jam containing stevia sweeteners with optimal quality at commercial scale.

## Supporting information

Data S1

## ENDNOTE

^1^
*F* = V × 0.01. Each milliliter of solution in a balloon is one liter equivalent to 0.01 grams of sugar.
